# Comparison of Current Methods for Signal Peptide Prediction in Phytoplasmas

**DOI:** 10.3389/fmicb.2021.661524

**Published:** 2021-03-25

**Authors:** Christophe Garcion, Laure Béven, Xavier Foissac

**Affiliations:** INRAE, Univ. Bordeaux, Biologie du Fruit et Pathologie, UMR 1332, Villenave d’Ornon, France

**Keywords:** bacteria, Mollicutes, SignalP, TMHMM, Phobius, secretion, effector, transmembrane domain

## Abstract

Although phytoplasma studies are still hampered by the lack of axenic cultivation methods, the availability of genome sequences allowed dramatic advances in the characterization of the virulence mechanisms deployed by phytoplasmas, and highlighted the detection of signal peptides as a crucial step to identify effectors secreted by phytoplasmas. However, various signal peptide prediction methods have been used to mine phytoplasma genomes, and no general evaluation of these methods is available so far for phytoplasma sequences. In this work, we compared the prediction performance of SignalP versions 3.0, 4.0, 4.1, 5.0 and Phobius on several sequence datasets originating from all deposited phytoplasma sequences. SignalP 4.1 with specific parameters showed the most exhaustive and consistent prediction ability. However, the configuration of SignalP 4.1 for increased sensitivity induced a much higher rate of false positives on transmembrane domains located at N-terminus. Moreover, sensitive signal peptide predictions could similarly be achieved by the transmembrane domain prediction ability of TMHMM and Phobius, due to the relatedness between signal peptides and transmembrane regions. Beyond the results presented herein, the datasets assembled in this study form a valuable benchmark to compare and evaluate signal peptide predictors in a field where experimental evidence of secretion is scarce. Additionally, this study illustrates the utility of comparative genomics to strengthen confidence in bioinformatic predictions.

## Introduction

Phytoplasmas are bacterial phloem-limited pathogens transmitted by insect vectors that generate diseases in infected plants. A large number of crops as well as ornamental plants and trees can be affected, showing altered development and yield losses (Lee et al., 2000). Phytoplasmas belong to the class Mollicutes, that derives from a Gram-positive, *Clostridium*- or *Lactobacillus*-related ancestor, but whose members are distinguished by the absence of a cell wall and are delimited by their plasma membrane (Lee et al., 2000; Hogenhout et al., 2008). Within Mollicutes, phytoplasmas are rather distant from mycoplasmas, and their closest relatives are bacteria from the *Acholeplasma* branch. Currently axenic cultivation methods of phytoplasmas are not yet available despite numerous attempts. As a consequence, many aspects of the biology of phytoplasmas are still to be explored. *In silico* analyses of phytoplasma genomes allowed to identify putative virulence factors, and three phytoplasma effectors (defined as proteins produced by pathogens that alter the physiology of the host) and their homologs have been the focus of several recent studies. Tengu is a short protein that induces dwarfism and witches’ broom, together with a downregulation of the jasmonic acid and auxin pathways (Hoshi et al., 2009; Minato et al., 2014). Remarkably, it was also shown to suppress induced cell death (Wang et al., 2018b) and to be processed by host proteases (Sugawara et al., 2013). The effector SAP11 impacts the development of the host plant and enhances its capacity to support reproduction of the insect vector through modulation of the jasmonate pathway (Sugio et al., 2011). SAP11 and its homologs were found to interact with specific transcription factors of the host (Sugio et al., 2011; Janik et al., 2017; Chang et al., 2018; Wang et al., 2018c; Pecher et al., 2019). Other reports indicated that SAP11 was also involved in interfering with the immune system and metabolic responses of the host plant (Lu et al., 2014b; Tan et al., 2016). Importantly, immunolocalization experiments showed that Tengu and SAP11 were found in plant tissues other than the phloem sieve tubes where phytoplasmas are confined, confirming that these effectors are secreted by phytoplasmas and then taken up by host sink tissues (Bai et al., 2009; Hoshi et al., 2009). SAP54 and PHYL1 are two homologous phytoplasma effectors that induce spectacular transformations of floral parts into leaf-like organs through destabilization of MADS-box transcription factors of the host (MacLean et al., 2011, 2014; Maejima et al., 2014, 2015; Kitazawa et al., 2017). For simplicity, in the following the SAP54/PHYL1 genes will be referred to as SAP54. Detailed molecular structures for SAP54 and its homologs are now available (Iwabuchi et al., 2019; Liao et al., 2019). Amazingly, it seems that a major role for SAP54 is to promote the attraction of insect vectors by the host plant (Orlovskis and Hogenhout, 2016). Phytoplasma effectors other than Tengu, SAP11, SAP54 have also been shown to interfere with the immune system of the host plant. Notably, it was established that part of the coding sequence of PM19_00185 from ‘*Candidatus* (*Ca.*) Phytoplasma mali’ induced susceptibility of *Arabidopsis thaliana* to *Pseudomonas syringae pv tabaci*, likely through a E3 ubiquitin ligase activity (Strohmayer et al., 2019). The SWP11 gene product from wheat blue dwarf phytoplasma induced cell death in *Nicotiana benthamiana*, and strikingly, the SWP12 and SWP21 (Tengu) proteins were able to counteract this process (Wang et al., 2018b). As effectors appear to be major players in the interaction with the host plant, their identification through genome mining stands as a prerequisite for a better understanding of phytoplasma diseases.

Identification of phytoplasma effectors only through sequence similarity is unlikely to succeed as they do not seem to be shared with other pathogens (Bai et al., 2009). However, the requirement to cross the plasma membrane to interact with host components leads to the detectable presence of features associated with secretion. A single secretion system is presumed to operate in phytoplasma cells. The initial finding of genes encoding essential components of the Sec translocation system in ‘*Ca.* P. asteris’ strain OY supported the existence of a functional Sec system in phytoplasmas (Kakizawa et al., 2001). This evidence was consistent with the cleavage of a N-terminal signal peptide observed for the antigenic membrane protein Amp (Barbara et al., 2002; Kakizawa et al., 2004). The analysis of other phytoplasma genomes indeed confirmed that they encode a Sec-dependent secretion system (Hogenhout et al., 2008; Kube et al., 2012). In *Escherichia coli*, the SecY, SecE, and SecG gene products assemble into a membrane-integrated protein conducting channel (Tsirigotaki et al., 2017). The *secG* gene appears to be absent in phytoplasmas (Kube et al., 2012), but it may be dispensable (Akimaru et al., 1991; Kube et al., 2014). From studies in *E. coli*, two pathways guide the Sec substrates to the membrane pore (Cranford-Smith and Huber, 2018). In the first one, a so-called signal recognition particle encoded by the *ffh* gene binds to the N-terminal sequence constituting the signal peptide of the nascent translation product emerging from the ribosome. It then docks to the FtsY protein, which ultimately allows the direct transfer of the polypeptide to the translocon. The ribosome provides the driving force that feeds the elongating polypeptide into the pore. The presence of conserved *ftsY* and *ffh* genes in genome sequences strongly suggest that this pathway also occurs in phytoplasmas (Kube et al., 2012). The second pathway is uncoupled from translation by ribosomes. In that case, chaperones such as trigger factor and SecB bind to the synthesized polypeptides and keep them in an unfolded state. The substrates are routed to or bound by SecA, which then acts as an ATP-dependent motor that pumps the substrate into the translocon. The SecA gene product was detected in multiple phytoplasmas (Wei et al., 2004). Unlike s*ecA* and trigger factor, the *secB* gene is not found in phytoplasma genomes, but other chaperones like GroEL/GroES or DnaK and DnaJ may functionally replace it, based on studies in *E. coli* (Siewert et al., 2014a). This second pathway is therefore also very likely to be functional in phytoplasmas. It is thought that the translation-uncoupled pathway allows to speed up the translocation process, as it is not limited by the ribosome elongation speed (Cranford-Smith and Huber, 2018). The pathway taken by each substrate depends upon the hydrophobicity of its N-terminal signal sequence (Tsirigotaki et al., 2017).

The signal sequences that allow recognition of the substrates to be translocated by the Sec machinery are typically located at the N-terminal end of the polypeptide. They are variable in sequence but share a common structure composed of a positively charged stretch of residues (*n*-region), a hydrophobic core (*h*-region) and a polar C-terminal domain containing the cleavage site (*c*-region) (Natale et al., 2008). The interaction with the Sec machinery elements is realized at the level of the *n*- and *h*-region, and modification of the charge, length, hydrophobic density or alpha-helix propensity of the different domains can impact the secretion outcome (Adams et al., 2002; Natale et al., 2008; Freudl, 2018). The presence of a hydrophobic region within signal peptides explains why they can be confused with transmembrane regions by prediction software, and vice-versa. Indeed, signal peptides can even be converted into N-terminal transmembrane regions by increasing the length of the h-region (Nielsen et al., 2019). The link between signal peptides and transmembrane domains is further reinforced by the fact that the translocation activity of the Sec machinery is required for correct insertion of membrane proteins into the plasma membrane. Indeed a lateral gate in the membrane pore allows translocating transmembrane domains to be released (Kuhn et al., 2017). The YidC insertase, also found in phytoplasmas, may assist the Sec machinery for this task, or work alone in the same purpose (Kuhn et al., 2017). After the translocation, the signal peptide is cleaved by a signal peptidase and then degraded by a signal peptide peptidase (Kim et al., 2008; Saito et al., 2011). Two types of signal peptidase have been described. Type I signal peptidase recognizes an A-X-A consensus sequence in its substrates and cleaves off the signal peptide (Paetzel, 2014), whereas type II signal peptidase catalyzes the cleavage of diacyl-glycerol-modified substrates, leading to the release of lipoproteins (Narita and Tokuda, 2017). The type II pathway seems to be missing in phytoplasmas, as discussed in the present work.

The identification of putative effectors from phytoplasma genomes thus relies on the detection of signal peptides and subsequent evaluation of the obtained hits. Different software packages among the most popular and efficient ones have been used in previous studies: SignalP 3.0 HMM (Bai et al., 2009; Saccardo et al., 2012; Chen et al., 2014; Music et al., 2018; Wang et al., 2018a), SOSUI and SignalP (Hoshi et al., 2009), SignalP 4.0 (Chung et al., 2013), SignalP 3.0 and 4.1 (Anabestani et al., 2017), SignalP 5.0 (Cho et al., 2019, 2020), Phobius (Kube et al., 2012, 2014; Siewert et al., 2014b; Quaglino et al., 2015), Phobius and SignalP 3.0 HMM (Sparks et al., 2018). SignalP in its first version was available online almost 25 years ago (Nielsen et al., 1997) and has been regularly updated and improved. SignalP 2.0 featured a hidden Markov model (HMM) along with artificial neural networks. SignalP 3.0 was released in 2004 and showed improved performance due to correction of errors in training sets, modification of the neural network design, and creation of a new score to discriminate between signal peptide and non-signal peptide sequences (Bendtsen et al., 2004). In SignalP 4.0 the HMM part was removed, and two neural networks were made available, including one that was trained with transmembrane sequences as negative data to improve the discrimination between signal peptides and transmembrane domains (Petersen et al., 2011). SignalP 4.0 was updated to 4.1 when a greater choice of options was allowed (Nielsen, 2017). Compared to SignalP version 4, SignalP 5.0 benefits from an internal algorithm more suited to signal peptides, a modified output score, and training on a dataset grouping (rather than separating) sequences from Eukarya, Gram-negative bacteria, Gram-positive bacteria, and Archaea (Almagro Armenteros et al., 2019). SignalP 5.0 also simultaneously differentiates Sec and Tat (twin-arginine translocation pathway, unidentified in phytoplasmas so far) substrates without having to rely on specialized separated software. Benchmark tests indicated that incremental versions of SignalP showed superior performance to previous versions, with the notable exception that SignalP 5.0 ranked second after SignalP 4.1 regarding the prediction of type I signal peptides (i.e., cleaved by type I signal peptidase) in Gram-positive bacteria (Almagro Armenteros et al., 2019). Phobius was released in 2004 and is based on a HMM. It was designed with an emphasis on the separation of transmembrane domains and signal peptides, and is available online (Käll et al., 2004, 2007). An important point is that the training datasets of SignalP 4.0 (and presumably of other versions) did not include sequences from *Mycoplasma* and related genera (Petersen et al., 2011). On the other hand, Bai et al. (2009) showed that SignalP 3.0 efficiently detected a signal peptide in a set of Mollicutes proteins. It therefore appeared of special interest to determine how these software products perform on phytoplasma sequences.

In this work, we compared the prediction results of these signal peptide predictors, in order to enable informed decisions when mining phytoplasma genome sequences for candidate effectors. We restricted our study to software and configurations already used in previous reports about phytoplasma effector analyses (i.e., SignalP versions 3.0, 4.0, 4.1, 5.0, and Phobius), with the aim to provide a reasonably thorough presentation of the results, and included the TMHMM software for comparison. We examined prediction results for all publicly available phytoplasma sequences that code for the documented effectors Tengu, SAP11, and SAP54 and homologs, for substrate-binding proteins, putative effectors, and specific membrane proteins.

## Materials and Methods

Identification of the phytoplasma homologous sequences was performed using the BLASTP software (Camacho et al., 2009) against the ‘non-redundant’ database (NCBI Resource Coordinators, 2018) with default parameters at the NCBI website. For the SAP54 dataset, the sequences from the phyl-B group of Iwabuchi et al. (2020) were excluded as they did not show the phyllody inducing phenotype observed with other members, although they may still have a functional signal peptide and yet-to-discover functions. For Amp and Imp, that can be highly variable, we first extracted from draft or complete phytoplasma genomes the coding sequences located between *groEL* and *nadE*, and *DnaD* and *PyrG* respectively. We then used the translated sequences as BLASTP queries to retrieve the full dataset of Amp and Imp homologous sequences. To ensure that our dataset was as exhaustive as possible, a keyword search (“antigenic membrane protein phytoplasma” and “imp” respectively) was also performed at Genbank, and validated hits from both strategies were merged.

For all datasets, sequences that were obviously truncated in N- or C-terminal, or exceeding the expected size, were removed from the final sets of hits. Sequence alignments were built with either Muscle 3.8.31 (Edgar, 2004) or Clustal Omega 1.1.0 (Sievers et al., 2011). Alignments were visualized and formatted with Seaview 5.0.4 (Gouy et al., 2010) and Jalview 2.11.1.0 (Waterhouse et al., 2009). Pred-Lipo (Bagos et al., 2008) and LipoP 1.0 (Juncker et al., 2003) were queried online at http://bioinformatics.biol.uoa.gr/PRED-LIPO and http://www.cbs.dtu.dk/services/LipoP respectively. The eulerr package 6.0.0 (Larsson, 2020) was used to draw initial Euler diagrams that were then adapted. Color schemes used in Figures 2, 4 have been elaborated by Paul Tol^1^.

The parameters used for the various versions of SignalP were set following common practice reported for phytoplasma sequences. SignalP 3.0 (Nielsen and Krogh, 1998; Bendtsen et al., 2004) was configured as described in Bai et al. (2009). Thus, in the present study, “SignalP3HMM” refers to the use of the online version of SignalP 3.0 at https://services.healthtech.dtu.dk/service.php?SignalP-3.0, using the following parameters: organism group: “Gram-positive bacteria”; method: “Hidden Markov Models,” Truncation: “Truncate each sequence to max. 70 residues” (default). Predictions with a Sprob score greater than or equal to 0.5 (default) were considered as signal peptide predictions, independently of the position of the predicted cleavage site. The neural network of SignalP 3.0 was not used. SignalP 4.0 (Petersen et al., 2011) was run through the online version at http://www.cbs.dtu.dk/services/SignalP-4.0/. In this study, “SignalP4.0” refers to SignalP 4.0 configured with the parameters indicated by Chung et al. (2013): organism group: “Gram-positive bacteria”; method: “Input sequences do not include TM regions”. Only proteins that were indicated as putatively secreted (“?” column) based on the default threshold of 0.57 for D score were considered to have a positive prediction of signal peptide. Similarly, “SignalP4.1sensitive” refers to the SignalP 4.1 software run online^2^ with parameters indicated in Anabestani et al. (2017): organism group: “Gram-positive bacteria”; *D*-cutoff values: “sensitive,” method: “Input sequences do not include TM regions.” According to the manual, SignalP 4.1 is the same package as SignalP 4.0 except that some formatting options were added. The “sensitive” option adjusts the *D*-cutoff value at 0.42 instead of 0.57. The predicted locations of signal peptide cleavage site were taken from the second “pos” column (Ymax score). “SignalP5” designates SignalP 5.0 (Almagro Armenteros et al., 2019) run online at https://services.healthtech.dtu.dk/service.php?SignalP-5.0 with the organism group set as “Gram-positive.” The TMHMM v2.0 software (noted as “TMHMM” in this study) (Krogh et al., 2001) was used locally or online at https://services.healthtech.dtu.dk/service.php?TMHMM-2.0, leaving the option “Use old model (version 1)” unchecked. Phobius (Käll et al., 2004, 2007) was run online at http://phobius.sbc.su.se/. Phobius can predict the presence of either a signal peptide or a transmembrane domain in protein sequences. This dual output was exploited separately. “Phobius_SP” indicates that only the signal peptide prediction (positive or negative) was considered. For “Phobius_SP_TM,” the prediction was taken as positive if either a signal peptide was predicted, as in Phobius_SP, or a transmembrane segment was predicted within the first 50 amino acids of the sequence.

Signal peptide groups used for redundancy removal in prediction counts were established based on signal peptide cleavage sites as predicted by SignalP 4.1. We took care to check that the various predictions were identical for identical signal peptides, and only very few exceptions were found: BAD04276.1 and WP_069028310.1 from the DppA family for SignalP3HMM predictions (data not shown), and the accessions mentioned in Figure 5. In such cases, the sequences were split into two subgroups and each subgroup was then considered as a group.

To build the phytoplasma gene families, the get_homologues software was used (Contreras-Moreira and Vinuesa, 2013). Phytoplasma genomes downloaded from Genbank were provided as input to get_homolog using the “-G -t 0” options and leaving all other options as default.

## Results and Discussion

### Predictions for Tengu, SAP11, and SAP54 Homologs

**FIGURE 1:**
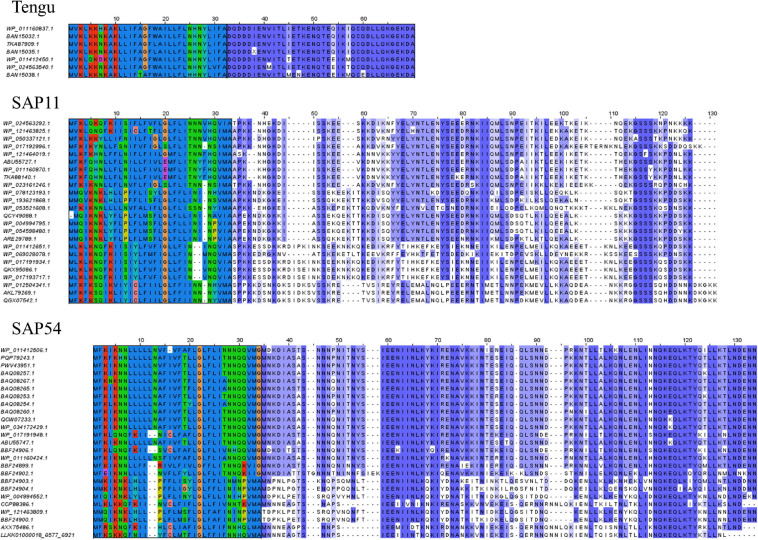
Sequence alignments of the Tengu, SAP11, and SAP54 homologs. The signal peptide regions have been colored according to the biochemical properties of amino acids (red: positively charged residues, blue: hydrophobic residues, green: polar residues, dark pink: negatively charged residues). Mature protein residues have been colored according to amino acid conservation among sequences (violet shades).

Up to now only the Tengu and SAP11 phytoplasma effectors have been demonstrated to be translocated and released extracellularly (Bai et al., 2009; Hoshi et al., 2009). In order to confidently increase the number of signal peptide sequences that could be used to assess the efficiency of signal peptide predictors on phytoplasma sequences, we took advantage of the availability of putative homologs and detailed functional studies. For each of the Tengu, SAP11, and SAP54 effectors, we collected respectively 7, 24, and 25 putative homologous sequences using the BLASTP software (Figure 1 and Supplementary Material M1). Because some of these sequences are shared among several phytoplasma strains, they derive from higher numbers of strains: respectively 22, 33, and 47 strains (Supplementary Tables 1–3). All effector homologs display a putative signal peptide with the expected features, i.e., a positively charged *n*-region, followed by a hydrophobic core and a *c*-region that contains the cleavage site. Interestingly, a large proportion of the mature protein sequences of these homologs have already been subjected to some functional tests validating their activity, for instance phenotyping after *in planta* expression or impact on expected targets: 6/7 (85.7%) for Tengu; 13/24 (54.2%) for SAP11; 14/25 (56%) for SAP54 homologs (Tables 1–3). This is due to the increasing number of publications devoted to the characterization of phytoplasma effectors, notably regarding their sequence diversity (Sugawara et al., 2013; Maejima et al., 2014; Chang et al., 2018; Iwabuchi et al., 2020; see a complete list of references in Supplementary Tables 1–3).

**TABLE 1:** Tengu homologs used in this study.

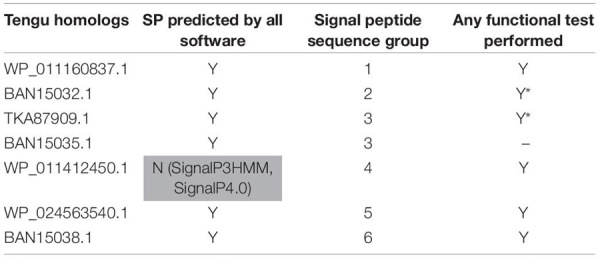

The second column indicates if a signal peptide is predicted by all of the SignalP3HMM, SignalP4, SignalP4.1sensitive, Phobius_SP, Phobius_SP_TM software (mention “Y” = yes). If not, cells were highlighted in gray (for better readability) with a “N” (no), and predictors that did not produce a positive prediction are mentioned. The third column shows the signal peptide group based on predictions of SignalP4.1sensitive (identical numbers are attributed to identical sequences). Finally, the last column mentions the presence of available evidence of effector activity (any functional test) from literature with the mention “Y”; * cases where the sequence of the mature protein is identical to another accession for which there is available data (see Supplementary Tables 1–3 for references).

**TABLE 2:** SAP11 homologs used in this study.

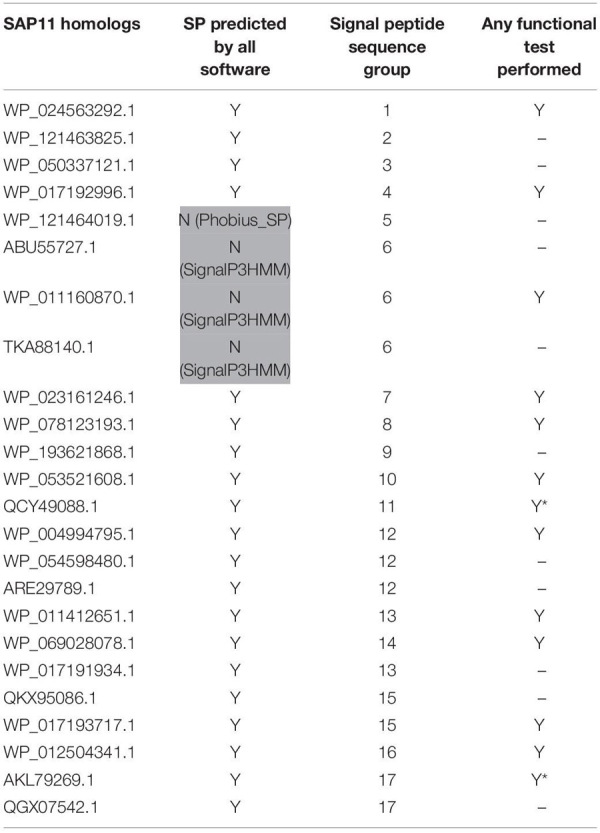

*Column content is described in the caption of Table 1.*

**TABLE 3:** SAP54 homologs used in this study.

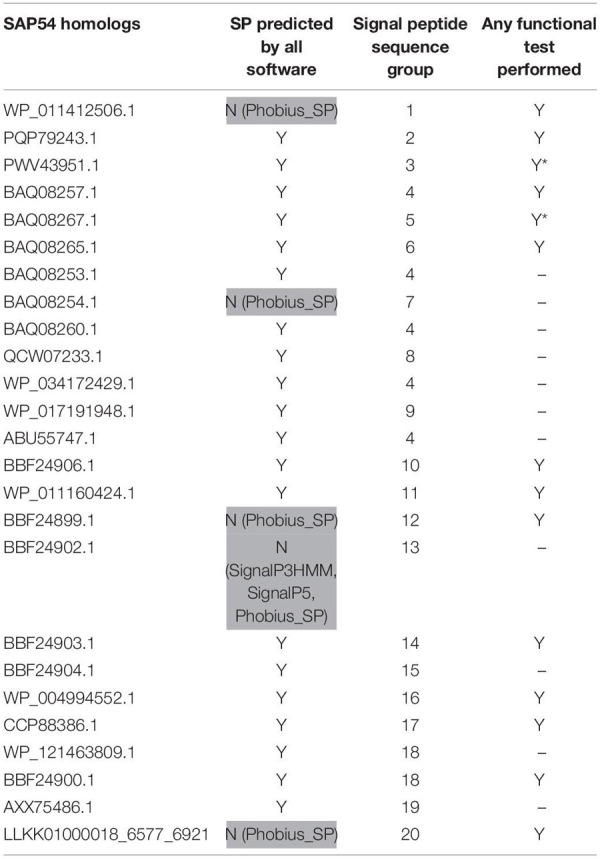

*Column content is described in the caption of Table 1.*

Based on sequence similarity and functional evidence, we hypothesized that these homologous sequences contain a functional signal peptide. These sequence sets thus constitute a benchmark to evaluate the performance of signal peptide predictors on phytoplasma sequences. However, although there were no duplicates among our homologous sequences (7 + 24 + 25 = 56 sequences in total), some had identical signal peptides (“signal peptide sequence group” in Tables 1–3). We took care to count only non-redundant signal peptide sequences when summarizing the various predictions in Table 4 (33 unique signal peptide sequences in total for accessions associated with functional data, or 43 for all homologs). Accessions with identical signal peptides were checked to have identical predictions. We compared the various flavors of the SignalP software used in phytoplasma effector studies (SignalP versions 3.0, 4.0, 4.1, and 5.0), as well as Phobius (signal peptide prediction referred to as “Phobius_SP” in this study). For SignalP version 4 we collected predictions using either the default thresholds (mentioned as “SignalP4.0”), or the ‘Sensitive’ 4.1 cut-off values (see section “Materials and Methods”). Given that both signal peptide and transmembrane segments share the presence of a hydrophobic core, predictions performed by the TMHMM software package (devoted to detection of transmembrane segments) were also included, as well as the cumulated predictions of signal peptide and transmembrane domain by Phobius (see section “Materials and Methods”; indicated by “Phobius_SP_TM” in the text). In both cases, prediction of a transmembrane domain in the 50 N-terminal amino acids was interpreted as a possible signal peptide.

**TABLE 4:** Count of positive signal peptide predictions of the protein sequences homologous to Tengu, SAP11, and SAP54.

	Homologs with functional tests				All homologs			
	Tengu	SAP11	SAP54	Total	Tengu	SAP11	SAP54	Total
Unique signal peptides	6	13	14	33 (100%)	6	17	20	43 (100%)
SignalP3HMM	5	12	14	31 (94%)	5	16	19	40 (93%)
SignalP4.0	5	13	14	32 (97%)	5	17	20	42 (98%)
SignalP4.1sensitive	6	13	14	33 (100%)	6	17	20	43 (100%)
SignalP5	6	13	14	33 (100%)	6	17	19	42 (98%)
TMHMM	6	13	14	33 (100%)	6	17	20	43 (100%)
Phobius_SP	6	13	11	30 (91%)	6	16	15	37 (86%)
Phobius_SP_TM	6	13	14	33 (100%)	6	17	20	43 (100%)

*Sequence redundancy at the N-terminus was addressed by counting only unique signal peptide sequences (each signal peptide sequence is associated with a signal peptide group as indicated in Tables 1–3).*

The prediction results for effector homologs with functional evidence readily point to some potential shortcomings for specific predictors (Table 4). Indeed, not all of the sequences were predicted to contain a functional signal peptide by all predictors. The performance of the various predictors differed depending on the homologous sequence set, revealing sequence-dependent sensitivity. For the Tengu dataset, the accession WP_011412450.1 that was not detected by SignalP3HMM and SignalP4.0 is present notably in the AYWB (aster yellows witches’ broom) phytoplasma genome (Table 1 and Supplementary Table 1). Consistently, this sequence was not listed as a putative effector in the original publication on AYWB effectors (Bai et al., 2009), which relied on SignalP 3.0 HMM. Available evidence suggest that the corresponding mature protein is active *in planta* (Sugawara et al., 2013). In the SAP11 dataset, SignalP3HMM also did not detect a putative signal peptide for three related sequences, unlike all other predictors. The associated mature protein from onion yellows strain OY-M was demonstrated to induce a bushy phenotype and to destabilize expected targets (Chang et al., 2018). Regarding the SAP54 dataset, Phobius_SP predicted a signal peptide in only 11 out of 14 sequences. However, for the remaining three sequences, that originate from AYWB phytoplasma, Japanese hydrangea phyllody phytoplasma, and Vc33 phytoplasma, Phobius predicted a N-terminal transmembrane domain instead of a signal peptide. This suggests that transmembrane segment identification could provide, in some cases, useful indication of putative signal peptides. This is independently confirmed by the fact that TMHMM identified a putative transmembrane segment for all sequences of the datasets.

If homologs without functional evidence are included in the analysis, further cases of discrepancies between predictors are revealed. Phobius_SP detected a signal peptide for 86% of unique N-terminal sequences, followed by SignalP3HMM (93%), SignalP4.0 and SignalP5 (98%) (Table 4). Predictions from SignalP4.1sensitive, TMHMM, Phobius_SP_TM suggested the presence of a signal peptide in all (100%) of the sequences. Only two accessions were predicted to be devoid of a signal peptide by at least two predictors: WP_011412450.1 (Tengu dataset) and BBF24902.1 (SAP54 dataset). A common theme between these two sequences is the presence of a negatively charged residue in the n-region of the putative signal peptide (Figure 1). The three sequences from the SAP11 dataset with a negative prediction by SignalP3HMM also showed a negatively charged residue within the *h*-region. Such residues may prompt signal peptide predictors to predict an absence of signal peptide, even though counter-examples can be found, consistently with the global charge of the *n*-region being likely more relevant than the simple presence of negatively charged residues. Another interesting point is the presence of two gene copies encoding SAP11 homologs in the genomes of ‘*Ca*. P. ziziphi’ and ‘*Parthenium hysterophorus*’ phyllody phytoplasma. In contrast to two periwinkle leaf yellowing phytoplasma strains and one onion yellows strain that also possess two *SAP11* copies (Cho et al., 2019), both homologs from each of them possess a putatively functional signal peptide. Finally, it should be emphasized that both SAP11 and SAP54 contain a SVM (Sequence Variable Mosaic) motif (pfam 12113) (Jomantiene et al., 2007). This motif is presumed to encode a signal peptide and seems to be well recognized by signal peptide predictors in general.

### Predictions for SBP Genes

In their seminal paper, Bai et al. (2009) emphasized that the detection of signal peptides in sequences similar to solute-binding proteins (SBP) strengthened the signal peptide predictions. Indeed, SBP are components of ATP-binding cassette (ABC) transporters that bind the substrate to be translocated. In bacteria, SBP are usually located in the periplasm (Gram-negative bacteria) or tethered to the membrane as lipoproteins (Gram-positive bacteria) and thus usually possess a signal sequence allowing translocation across the plasma membrane. In order to evaluate the performance of signal peptide predictors on SBP sequence datasets, we used the BLASTP software to identify phytoplasma sequences similar to the AYWB SBPs described in Bai et al. (2006), i.e., NlpA, ArtI, DppA, MalE, ZnuA, PotD. Among them the *potD* gene products contain a C-terminal transmembrane domain and therefore have a different predicted topology, so we chose to not include them in our study. We could collect at least one sequence from all deposited phytoplasma genomes, thus representing the widest possible phylogenetic diversity, even if some groups are more represented than others (Supplementary Material M1 and Supplementary Table 4). We manually removed sequences that were obviously truncated, and followed the same process as for Tengu, SAP11, SAP54 homologs by counting signal peptide predictions for each of the different predictors after removal of duplicated signal peptide sequences (Table 5).

**TABLE 5:** Count of positive signal peptide predictions for each SBP family.

	NlpA	ArtI	DppA	MalE	ZnuA	Total
Unique signal peptides	14	9	30	18	20	91 (100%)
SignalP3HMM	8	5	16	13	12	54 (59%)
SignalP4.0	14	9	28	14	18	83 (91%)
SignalP4.1sensitive	14	9	30	18	20	91 (100%)
SignalP5	1	0	2	9	10	22 (24%)
TMHMM	14	9	30	18	20	91 (100%)
Phobius_SP	8	3	2	3	4	20 (22%)
Phobius_SP_TM	14	9	30	18	20	91 (100%)

*Like in Table 4, only unique signal peptides are counted.*

**FIGURE 2:**
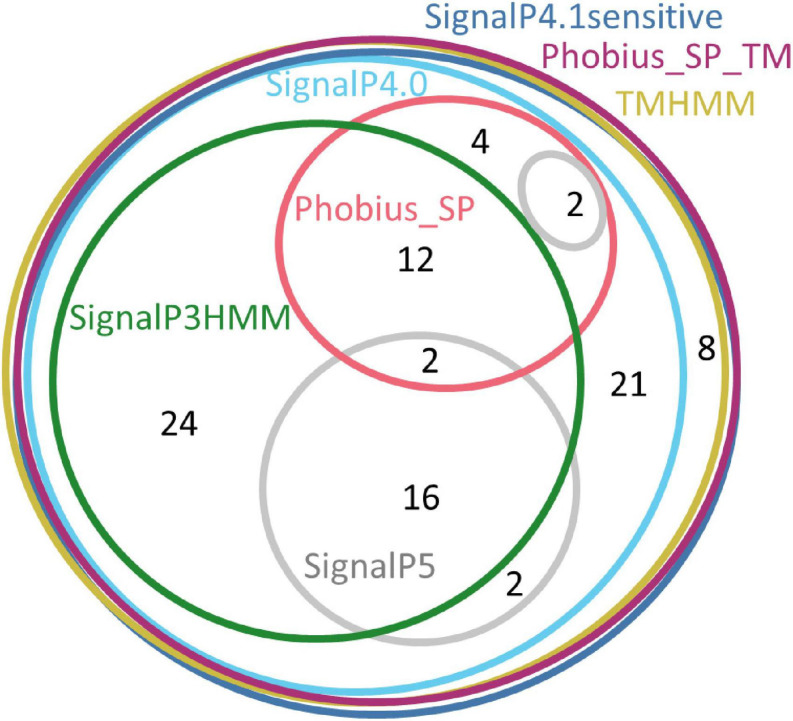
Euler diagram representing the overlap of positive signal peptide predictions between predictors for all SBP sequences. The numbers indicate counts of sequences. Each software package has been associated with a specific color. As it was not possible to produce a strict Euler diagram, the SignalP5 ellipse was split into two parts.

Only three software packages suggested the presence of a signal peptide for the totality of the 91 sequences: SignalP4.1sensitive, TMHMM, and Phobius_SP_TM. For the other software products, the predictions ranged from 22% (Phobius_SP) to 91% (SignalP4.0). The current latest version of SignalP, SignalP 5.0, detected a signal peptide only in 24% of these sequences. The sequences that were predicted to have a signal peptide varied depending on the predictor, showing that each predictor has its own specificity (Figure 2).

**FIGURE 3:**
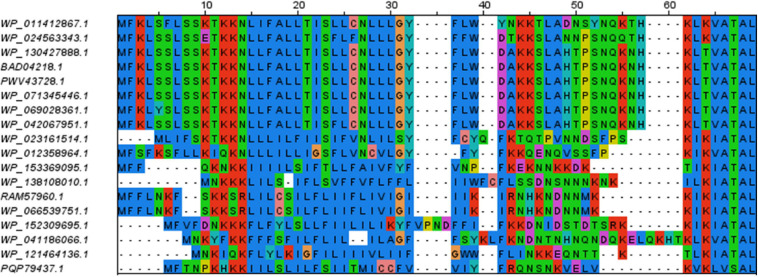
Sequence alignment of the N-terminal residues of members of the NlpA family. The remaining of the alignment (C-terminal parts) is not shown. The color code is the same as in Figure 1. Positively charged residues and stretch of hydrophobic residues of the signal peptides are well conserved.

The SBP sequences were closely examined for the presence of a signal peptide and SBP features. Sequence alignments clearly suggested the presence of typical signal peptides with a short positively charged N-terminus followed by a hydrophobic stretch of amino acids (see Figure 3 for the example of NlpA protein sequences). We found only one group of exceptions where conserved SBP sequences from the NlpA family lacked a signal peptide and were not associated with an ABC-transporter operon (Supplementary Table 5). Each of the corresponding genes was located in tandem with another gene encoding a SBP devoid of a putative signal peptide. These gene products might be involved in intracellular signaling (Scheepers et al., 2016) and were not included in our analyses. We also examined carefully the sequences hits recovered by BLASTP but apparently lacking a complete signal peptide. They were excluded from this study, but were found to result from either one of the following artifacts: (i) N-terminal sequence is not available (i.e., incomplete genomic sequence), (ii) frameshifts disrupted the reading frame, but there is a reading frame encoding the missing part of the signal peptide, (iii) an alternative start codon was used for conceptual translation. In only two cases, we could not succeed in detecting a classical signal peptide (accessions PQP79209.1 and WP_050337100.1 from two different strains of ‘*Ca*. P. phoenicium,’ *znuA* gene family). In addition, the sequences identified by using BLASTP showed sequences signatures associated with SBP activity (accessions cl21456, cd00995, cl01709, or cl00262 of NCBI Conserved Domain Database). We confirmed that their encoding genes are located at close proximity (presumably within same operon) to other ABC transporter components, i.e., ATP-binding and permease subunits. Exceptions were *dppA* sequences of ‘*Ca*. P. oryzae,’ *Cynodon dactylon* phytoplasma, and sugarcane grassy shoot phytoplasma, that are isolated but with the corresponding operon being devoid of SBP gene (Supplementary Figure 1 and data not shown). Finally, a few SBP sequences of the DppA family were found to be duplicated or even triplicated within their operon, notably in the 16SrI and 16SrXII groups, explaining the higher number of sequences for this family (Supplementary Table 4). In some cases, such duplications events were associated with loss of typical signal peptide for one gene copy, but in all cases at least one copy with a typical signal peptide remained. The *dppA* gene family also showed the highest sequence diversity, as well as differences in the order of the subunits within the ABC transporter operon (Supplementary Figure 1). In summary, sequence analysis supports the presence of a genuine signal peptide in all selected SBP sequences (Supplementary Table 4). Even if no experimental data is available regarding the secretion of these phytoplasma SBPs, the presence of a secretion signal is consistent with the subcellular location required for their activity and what is generally observed in bacteria.

As mentioned above, SBP are usually found as lipoproteins in Gram-positive bacteria (van der Heide and Poolman, 2002). In that case, the preprotein is acylated by Lgt transferase activity on a specific cysteine residue that is part of a motif termed lipobox, and the signal peptide is cleaved by the type II signal peptidase LspA, leaving the modified cysteine residue as the first amino acid of the mature protein (Narita and Tokuda, 2017). However, it is unlikely to be the case for phytoplasmas. Bai et al. (2009) already noted that none of the AYWB *nlpA, artI, dppA, malE, znuA* gene products were predicted as a lipoprotein. We submitted the 115 phytoplasma SBP sequences validated in this study (Supplementary Table 4) to lipoprotein predictors Pred-Lipo and LipoP, and noticed that respectively none and 13 of them were predicted to contain a type II signal peptide (Supplementary Table 6). We believe that the positive type II predictions are artifacts due the presence of a cysteine located close to the cleavage site. The fact that this cysteine is not conserved, even in closely related sequences for some cases, suggests that it is not strictly required (example in Supplementary Figure 2). We think it unlikely that related phytoplasmas with similar SBP protein sequences would resort on different molecular mechanisms for secretion. Huang and Ho (2007) analyzed the *dppA* sequence of Loofah witches’ broom phytoplasma and suggested the presence of a lipobox. However, its position would imply an unusually long signal peptide of 54 residues and this motif is not conserved among the other phytoplasma *ddpA* sequences. Another element against the presence of lipoproteins in phytoplasmas is the apparent absence of *lgt* and *lspA* genes in phytoplasma genomes. We noted with interest that the situation is different for *Acholeplasma* sp., where most of the identified SBP are predicted to contain a type II signal peptide by Pred-Lipo and LipoP (Supplementary Table 7), and contain a typical lipobox (data not shown). In addition, *Acholeplasma* genomes possess readily detectable *lgt* and *lspA* genes, and protein acylation was demonstrated in this genus (Serebryakova et al., 2011). These observations raise interesting questions about the evolution of phytoplasmas from acholeplasma-like ancestors and how the transition from lipoprotein SBP to non-lipoprotein SBP occurred.

On a functional point of view, if phytoplasma SBPs are not lipoproteins, how does it happen that they do not diffuse away from phytoplasma cells when their peptide signal is cleaved? A hypothetical scenario could be that the signal peptide is not cleaved, acting as a membrane anchor. An alternative hypothesis with perhaps more experimental grounds is that the SBP remains bound to the ABC transporter subunits, whether it has captured a ligand or not. This has been shown to occur for the histidine transporter of *Salmonella typhimurium* (Ames et al., 1996).

### Predictions for Selected Putative Effector Families

In order to enlarge further our datasets containing signal peptides, we looked for phytoplasma gene families coding for putative effectors. Selection criteria were based on the number of members, presence within several phytoplasma phylogenetic groups, overall levels of conservation but also sequence variation in the region of the putative signal peptide. These criteria were designed to select for consistent families that allow to challenge signal peptide predictors and discriminate between them. We excluded sequences starting with a SVM motif. Among the few such gene families that were found, we selected three of them that were named after their member from AYWB (respectively AYWB_387, AYWB_376, and AYWB_042). The sequences and list of accessions used in this study are available in Supplementary Material M1 and Supplementary Table 8. Unlike AYWB_042, AYWB_387 and AYWB_376 had already been identified as containing a putative signal peptide and were also designated respectively as SAP08 and SAP09 (Bai et al., 2009).

**FIGURE 4:**
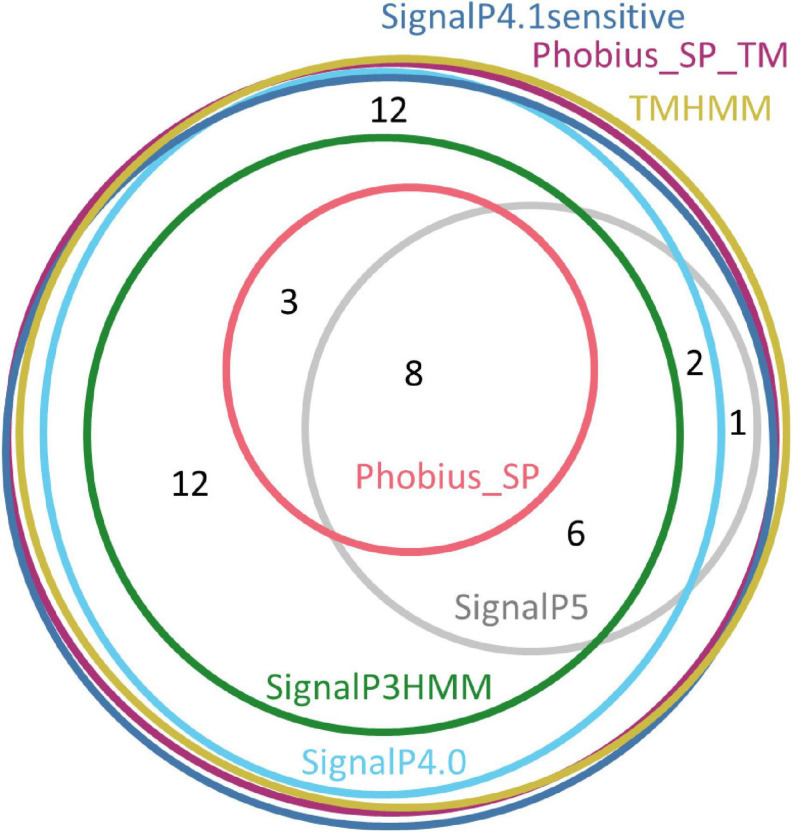
Euler diagram representing the overlap of positive signal peptide predictions for pooled sequences from the AYWB_387, AYWB_376, and AYWB_042 families. The numbers indicate counts of sequences.

In a similar process as above, we performed signal peptide predictions for each of the family members and counted the number of putative signal peptides detected by the various software packages (Table 6). Similarly to the Tengu/SAP11/SAP54 effectors and SBP sequences, only SignalP4.1sensitive, TMHMM and Phobius_SP_TM predicted that 100% (*n* = 44) of these sequences included a signal peptide. SignalP4.0 followed closely with 98%, and SignalP3HMM, SignalP5, and Phobius_SP respectively detected a signal peptide in only 66, 39, and 25% of the sequences. Again, the sequences that were predicted to be devoid of a signal peptide varied depending on the software (Figure 4). Only eight accessions were predicted to contain a signal peptide by all of the software packages. A major part (8 out of 11 – 73%) of positive predictions by Phobius_SP were also realized by all other software, the remaining (27%) being in contradiction with SignalP5 predictions. The cumulated positive predictions of SignalP3HMM, SignalP5 and Phobius_SP did not include the totality of the sequences (32 sequences out of 44 – 73%).

**TABLE 6:** Count of positive signal peptide predictions in the AYWB_387, AYWB_376, AYWB_042 families.

	AYWB_387	AYWB_376	AYWB_042	Total
Unique signal peptides	11	21	12	44 (100%)
SignalP3HMM	6	21	2	29 (66%)
SignalP4.0	11	21	11	43 (98%)
SignalP4.1sensitive	11	21	12	44 (100%)
SignalP5	5	9	3	17 (39%)
TMHMM	11	21	12	44 (100%)
Phobius_SP	4	6	1	11 (25%)
Phobius_SP_TM	11	21	12	44 (100%)

*Like in Tables 4, 5, only unique signal peptides are counted.*

A not-yet-solved question is whether the sequences of this dataset do include a functional signal peptide that is processed by the secretion machinery. We currently have no experimental evidence of secretion for any of them, but the conservation of positively charged amino-acids before a core of hydrophobic residues, which are typical features of signal peptides, provides support for this hypothesis (sequence alignments in Supplementary Figures 3–5). Incidentally, in most cases the genes of the AYWB_387 and AYWB_376 families are located close to PMU-associated genes in genomic sequences, with PMU being presumed effector-rich regions (Sugio and Hogenhout, 2012; Chung et al., 2013; Ku et al., 2013). For example, in AYWB genome, the *AYWB_387*, *AYWB_376*, and *SAP11* genes are included in a region of 11.2 kb that also features PMU elements (Bai et al., 2009). In some genome drafts, *AYWB_387* and *AYWB_376* homologs are located in short contigs whose ends could not be assembled, likely due to repeated sequences such as PMUs. The duplication of family members in some genomes (Supplementary Table 8) is also linked to their location in PMU regions.

**FIGURE 5:**
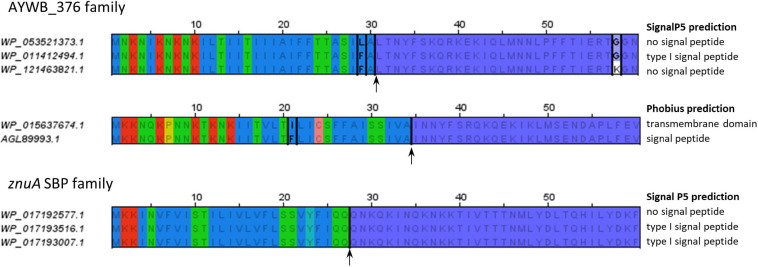
Similar N-terminal sequences with different signal peptide predictions. The alignments only show the first 60 residues of each sequence. Residues conserved within an alignment are written in gray; accession-specific residues are in black and boxed. The alignment of sequences from *znuA* gene family shows no sequence variation. Background colors follow the same color code as in Figure 1. Putative cleavage sites (according to SignalP5 or Phobius as required) are indicated by an arrow. Mentions on the right of the alignments indicate the respective predictions of SignalP5 or Phobius for each sequence.

The fact that some, but not all, of the homologs of AYWB_387, AYWB_376, and AYWB_042 were predicted to have a signal peptide depending on software packages, highlights the limits of signal peptide predictions and illustrates the differences between signal peptide predictors. Whatever the true secretion status of these gene products, the most consistent predictors within each family are SignalP4.1sensitive, TMHMM and Phobius_SP_TM. This dataset also provides examples of how predictors can be specifically affected by variations in primary sequence. We noticed that very similar N-terminal sequences sometimes resulted in different prediction outcomes, particularly with SignalP5, as shown in Figure 5. In the AYWB_376 family, the accessions WP_053521373.1 and WP_011412494.1 display a single amino acid variation within a hydrophobic stretch in their first 60 amino acids (leucine to phenylalanine at position 29), yet they have different SignalP5 prediction. A possible explanation could be an interference with cleavage site recognition, as cleavage is predicted at position 30. However, accessions WP_011412494.1 and WP_121463821.1 are identical on their first 57 residues, well over the whole putative signal peptide, but have different SignalP5 predictions, illustrating the high sensitivity of SignalP5. Another example from the same family shows that replacement of one hydrophobic amino acid by another within the hydrophobic core also leads to differing Phobius predictions, in that case transmembrane domain or signal peptide. This example shows how the prediction consistency is increased if N-terminal transmembrane domains are considered as a possible indication of a signal peptide (“Phobius_SP_TM” in Tables 4–6). In a last example from the ZnuA SBP family, accessions WP_017192577.1, WP_017193516.1, and WP_017193007.1 have identical N-termini for 62 residues but varying signal peptide predictions by SignalP5 (Figure 5). As the putative signal peptide is only 27 amino-acids, as predicted by SignalP5 itself, such a difference of prediction output is surprising. These particular cases suggest that the high sensitivity of SignalP5 resulting from its design and training set may provide excellent performance in general but might not be adapted to phytoplasma sequences.

### Predictions for Selected Membrane Proteins

As increased detection sensitivity can be associated with increased false positive risk, we challenged the evaluated predictors with sequences that resemble signal peptides. In order to collect such sequences, we looked for phytoplasma gene families satisfying several criteria. First, they should be widely conserved among phytoplasmas, allowing to observe a conserved trend and not to rely only on few particular sequences. Second, the sequences from this family should include a hydrophobic stretch of residues in their N-terminus, but, unlike typical signal peptide sequences, only rare positively charged amino acids in the *n*-region. Thus, in accordance with data from other studies (see below) and the positive-inside rule (Baker et al., 2017), these proteins presumably contain a N-terminal transmembrane domain that leaves the bulk of the protein in the cytosol, except a very short extracellular domain. As a consequence, these sequences can be used to evaluate the ability of signal peptide predictors to discriminate typical signal peptides and transmembrane domains. The accessions and sequence alignments of these four families are available in Supplementary Tables 9,10 and Supplementary Figures 6–9).

The first selected gene family encode RmuC, of which the precise function is unknown, but that has been associated with DNA recombination processes (Slupska et al., 2000). Intriguingly, the *RmuC* gene was detected only in 16SrI and 16SrXII phytoplasma groups, as briefly mentioned by Kube et al. (2012) and Saccardo et al. (2012), whereas it is readily detectable in other genera of the Mollicutes class, such as *Acholeplasma* and *Spiroplasma*, as well as many bacterial genera outside Mollicutes. The RmuC protein from *E. coli* is also predicted to contain a transmembrane domain at its N-terminus (Lomize et al., 2017). The second family focuses on the *YneF* gene, that codes for a short protein of about 70 amino acids of unknown function (uncharacterized protein family UPF0154, pfam PF03672). As *yneF* is widely conserved in phytoplasmas, but also in acholeplasmas and mycoplasmas, and in distant genera such as *Bacillus* that have a different lifestyle, it is likely to perform a conserved function. We had a similar case with the third family, referred to as AYWB_444 family. This family is shared among phytoplasmas but also with acholeplasmas and other bacterial taxons from Tenericutes. While its function is unknown, and no conserved functional domain has been identified, the conservation range suggests that it is involved in housekeeping functions. The last gene family is RNase Y, which has been involved in mRNA decay in *Bacillus subtilis* and other Gram-positive bacteria, and for which there is experimental evidence of association with the plasma membrane through a N-terminal membrane-spanning domain (Bechhofer and Deutscher, 2019; Hamouche et al., 2020). Similar sequences are found in phytoplasmas and acholeplasmas, and also in many Gram-positive bacterial taxons. We faced an ambiguity for RNase Y sequences from 16SrI ribosomal group and ‘*Ca.* P. australiense’ genomes, due to two alternative start codons generating protein sequences differing by respectively 11 and 18 amino acids (Supplementary Tables 9,10 and Supplementary Figure 9).

As above, predictions for the different signal peptide predictors were collected and summarized in Table 7. Large differences were observed between predictors, with none of the 68 sequences of the *RmuC*, *YneF*, *AYWB_444*, *RNaseY* families predicted to contain a signal peptide by Phobius_SP, and only 1% for SignalP5, whereas SignalP4.1sensitive counts reached close to 70%. These differences were similar at the level of individual gene families, and therefore suggestive of the respective tendency of predictors to consider this type of N-terminal transmembrane domains as putative signal peptides. It is important to note that the frequent occurrence of signal peptide prediction for these membrane proteins by SignalP version 4 is linked with the specific configuration used in this study for the ‘method’ parameter. SignalP version 4 has been designed with an improved capacity to distinguish signal peptides from transmembrane domains (Petersen et al., 2011). When this ability is switched on by using SignalP-TM network (method parameter set to “Input sequences may include TM regions”), only 1 sequence (WP_066539764.1) out of 68 was predicted to contain a signal peptide. This particular accession from ‘*Ca.* P. oryzae’ was also predicted to contain a signal peptide by SignalP3HMM and SignalP5, likely because of the presence of three lysine residues before the transmembrane domain, but not by Phobius_SP. Taken as a whole, comparison of Table 7 with Tables 4, 5 clearly illustrate that for the tested predictors, a gain in sensitivity was also associated with an increased risk of false positives when challenged with sequences partially similar to signal peptides.

**TABLE 7:** Count of positive signal peptide predictions for the *RmuC*, *YneF*, *AYWB_444*, *RNaseY* gene families.

	RmuC	YneF	AYWB_444	RNase Y	Total
Unique N-terminal sequences	7	21	21	19	68 (100%)
SignalP3HMM	1	1	1	1	4 (6%)
SignalP4.0	7	8	2	6	23 (34%)
SignalP4.1sensitive	7	16	12	9–12	44–47 (65–69%)
SignalP5	0	0	0	1	1 (1%)
TMHMM	7	21	21	19	68 (100%)
Phobius_SP	0	0	0	0	0 (0%)
Phobius_SP_TM	7	21	21	19	68 (100%)

*For RNaseY and total, a range of values is given, because there is some uncertainty on the translation start codon of some genes. Redundant N-terminal sequences were counted only once, as in Tables 4–6. Here, these N-terminal sequences were defined operationally as the regions identified as possible signal peptides by SignalP4.1sensitive, even if the signal peptide prediction score of these sequences did not reach the threshold value.*

### Predictions for Immunodominant Membrane Proteins

Unlike membrane proteins described above, immunodominant membrane proteins are phytoplasma membrane proteins for which the bulk of the protein is extracellular and only a very short portion is intracellular (Kakizawa et al., 2006), allowing them to interact with host molecules (Konnerth et al., 2016). They are associated with different gene products of various topologies. Among them, Amp was suggested to possess a cleaved signal peptide and a C-terminal transmembrane domain, based on experimental evidence (Barbara et al., 2002; Kakizawa et al., 2004). We therefore gathered available Amp sequences to generate another dataset of signal peptide-containing phytoplasma proteins. As Amp proteins are notoriously variable, we proceeded both by keyword search and BLASTP similarities to collect protein sequences. Hundred and eight Amp sequences were found in total, with a presence restricted to phytoplasma ribosomal groups 16SrI, 16SrXII, and 16SrXIII (sequences and accessions in Supplementary Material M1 and Supplementary Table 11). Prediction results by the different predictors after elimination of redundant sequences are shown on Table 8. All tested predictors, excepted Phobius_SP, unanimously detected a signal peptide in all Amp N-terminal sequences, thus achieving a prediction rate of 100%. Phobius_SP predicted a signal peptide for 64% of Amp sequences, while it identified a transmembrane domain in the remaining sequences. If this prediction is also considered as an indication of the presence of a signal peptide, as for Phobius_SP_TM, the prediction rate also reaches 100%. Thus, like for documented effectors and SBP, transmembrane domain predictors TMHMM or Phobius_SP_TM also allow suggestion of a putative signal peptide in all tested Amp sequences. The analysis of the cases predicted by Phobius to have a transmembrane region instead of a signal peptide suggests that replacement of a single amino acid of the presumed cleavage site leads Phobius to predict a transmembrane domain, whereas the various SignalP versions simply indicated another possible cleavage site located nearby. Alternatively, it is possible that increasing the hydrophobicity close to the cleavage site also promotes prediction of a transmembrane segment by Phobius.

**TABLE 8:** Count of positive signal peptide predictions for Amp and Imp sequences.

	Amp	Imp
Unique signal peptides or N-terminal sequences	22 (100%)	43 (100%)
SignalP3HMM	22 (100%)	16 (37%)
SignalP4.0	22 (100%)	12 (28%)
SignalP4.1sensitive	22 (100%)	43 (100%)
SignalP5	22 (100%)	0 (0%)
TMHMM	22 (100%)	43 (100%)
Phobius_SP	14 (64%)	0 (0%)
Phobius_SP_TM	22 (100%)	43 (100%)

*Redundant N-terminal sequences were counted only once, as in Tables 4–7.*

Other phytoplasma proteins have been described with a presumed similar topology: VmpA and VmpB from 16SrV group (Renaudin et al., 2015; Malembic-Maher et al., 2020), and Vmp1 from 16SrXII group (Cimerman et al., 2009). Available evidence strongly suggests that a signal peptide is cleaved from VmpA in phytoplasma cells (Renaudin et al., 2015). Only few full-length sequences could be collected by BLASTP for these proteins (Supplementary Material M1 and Supplementary Table 12). The comparison of predictions shows that for VmpA, only SignalP5 and Phobius_SP did not detect the signal peptide, whereas all predictors identified a signal peptide for VmpB and Vmp1 (Supplementary Table 12).

Another documented phytoplasma immunodominant membrane protein is Imp. Imp is anchored in the plasma membrane by a N-terminal transmembrane helix (Berg et al., 1999; Kakizawa et al., 2009; Neriya et al., 2011; Siampour et al., 2013). As for Amp, Imp sequences were retrieved using two different approaches based on sequence similarity and keyword searches. Hundred and twenty-three Imp sequences (Supplementary Material M1 and Supplementary Table 13), corresponding to 43 unique N-terminal sequences, were collected and found to originate from all branches of the phytoplasma phylogenetic tree. These sequences were submitted to the predictors tested in this study (Table 8). Results were highly contrasted, as SignalP5 and Phobius_SP detected a peptide signal in none of the sequences, SignalP4.1sensitive in all of the sequences, and SignalP3HMM and SignalP4.0 had positive predictions for 37 and 28% of the sequences respectively. The transmembrane predictors TMHMM and Phobius_SP_TM detected a transmembrane region in 100% of the sequences, thus constituting obvious cases of false positives if all positive predictions of transmembrane regions are taken as an indication of signal peptides. As the signal peptide predictors tested in this study have been designed to discriminate between transmembrane regions and signal peptides, for the Imp dataset, the best performance will be associated with the lowest number of positive predictions. Following this principle, regarding the Imp dataset, the best performance comes from SignalP5 and Phobius, whereas the worst performance is produced by SignalP4.1sensitive. However, similarly to the case of membrane proteins discussed above, it must be emphasized that another configuration of SignalP version 4 would lead to very different results. Indeed, if the SignalP-TM network is used, instead of the SignalP-noTM network used throughout this study, no Imp sequence is predicted to contain a signal peptide, again pointing to the major effect of this parameter in the configuration of SignalP version 4.

### Global Comparison of Signal Peptide Lengths and Scores

The availability of the various sequence datasets detailed in this study opens up the possibility to outline conserved properties and possible variations of phytoplasma signal peptides. For instance, in their search of AYWB putatively secreted proteins, Bai et al. (2009) focused on candidates with predicted signal peptides longer than 20 amino acids and shorter than 50 amino acids. How does that rule of thumb fit with the Tengu, SAP11, SAP54, SBP and Amp sequence datasets? The analysis of signal peptide length distribution for these datasets shows that, whatever the predictor, a large fraction of predicted signal peptide sequences displays a length between 30 and 35 residues, and that the 20–50 amino acid length range captures the whole diversity of these datasets (Supplementary Figure 10). Incidentally, the same type of analysis performed on various phytoplasma genomes revealed a similar distribution (data not shown). Notable exceptions came from SignalP4.1sensitive and Phobius, which both predicted signal peptides shorter than 20 amino acids, that belong for some of them to likely housekeeping gene products such as ribosomal proteins (data not shown). Thus, dismissing predictions of signal peptides shorter than 20 residues seems currently to be a reasonable assumption.

The various versions of SignalP also provide access to a score which allows the discrimination of signal peptides from non-signal peptides. This score can be interpreted as the confidence in the signal peptide prediction. Supplementary Figure 11 shows a comparison of these scores across the various datasets. For all the SignalP versions there is a global trend to output lower values with transmembrane protein datasets (RmuC, YneF, AYWB_444, RNAseY). However, scores with moderate values can be achieved by sequences from datasets of different status, suggesting that such indicator values may not be helpful for an uncharacterized gene product. Furthermore, the prediction score, as a confidence score, may indicate the relatedness to signal peptide sequences of the software training set, which might not be fully relevant in the case of phytoplasma sequences.

Finally, we also looked if there was any correlation between cleavage score values and the expected status of the sequence (signal peptide or transmembrane region) (Supplementary Figure 12). Like the predictions scores, we observed that cleavage scores were not highly informative, meaning that moderate values could correspond to sequences with either a signal peptide or a transmembrane domain.

## General Discussion and Conclusion

A major question addressed in this study is the identification of software packages best adapted to identify signal peptides from phytoplasma sequences. We could rely notably on sequences of documented effectors, SBP and membrane proteins from several gene families, intending to cover as much as possible of the phylogenetic diversity of phytoplasmas, and thus drawing on conserved biological features rather than isolated sequences. A primary conclusion is that there is no ideally performing software among those that were tested, since none of them simultaneously detects all expected signal peptides and ignores all N-terminal transmembrane domains. However, depending on the final purpose, some of them may appear more suited than others. Biologists seeking confidence in predictions, or instead sensitivity to avoid missing interesting candidates, might choose different options. We observed that SignalP 5.0 and Phobius_SP seemed particularly good at discriminating N-terminal transmembrane domains from signal peptides, however they missed many expected signal peptides. While they are both popular and highly cited software, a likely explanation for this failure in our case is the lack of experimentally proven phytoplasma signal peptide sequences that could be used for software training, whatever the efforts spent in software design. From the results we obtained, it seems that the strength of SignalP 5.0 and Phobius_SP lies in discrimination ability and confidence in their positive predictions, even if a more precise evaluation of this confidence would be required through challenges with many more sequences devoid of signal peptides. Moreover, the various software cannot be simply ranked by increasing order of confidence in positive predictions. Indeed, except for SignalP4.1sensitive, TMHMM and Phobius_SP_TM, predictions have only a partial overlap (Figures 2, 4), leaving room for uncertainty in the case of uncharacterized gene products.

Conversely, SignalP4.1sensitive, and obviously signal peptide suggestions derived from TMHMM and Phobius_SP_TM predictions, were confounded by transmembrane domains, but achieved 100% of detection of putative signal peptides. They were also more consistent in their predictions from putative effectors (Table 6). Such a success rate is appealing, even if it must be kept in mind that only a part of the sequence space of phytoplasma signal peptides has been tested here. These predictors might be more appropriate when attempting to build an exhaustive set of putatively secreted effector candidates from a phytoplasma genome, at the price of a background noise of false-positive proteins. The fact that all expected signal peptides were detected by transmembrane domain predictors also highlights the relatedness between phytoplasma signal peptides and transmembrane helical segments. Tests by the creators of TMHMM indicated that about 60% of signal peptides from Gram-positive bacteria were recognized as transmembrane domains, compared to 20% for sequences of eukaryotic and Gram-negative bacterial origins, probably because of the longer *h*-regions of signal peptides from Gram-positive bacteria (Krogh et al., 2001). Thus, it seems that phytoplasmas also share this feature with their Gram-positive ancestors, i.e., the h-regions of their signal peptides are similarly sized to alpha-helical transmembrane regions. In turn, this can be exploited by using transmembrane predictors such as TMHMM or Phobius_SP_TM to indicate the possible presence of signal peptides. Moreover, this explains why the SignalP4.1sensitive configuration used here (SignalP-noTM network) also detected 100% of tested signal peptides, as it is based on a neural network not trained with transmembrane sequences as negative data. Comparison with the other neural network available for SignalP 4.1 (SignalP-TM network) that discriminates transmembrane regions confirmed this conclusion (data not shown).

The close proximity between phytoplasma signal peptides and transmembrane domains may appear as a difficulty when looking for soluble proteins that could act as virulence factors. However, even if the current software packages reach their limits here, and data about relative numbers of secreted soluble proteins and membrane proteins are lacking at the moment, close examination of sequences might help in a few cases. Notably, alignment of the sequence of interest with similar sequences from other phytoplasmas could reveal conserved biological features and amino acid composition trends; the occurrence of a barely positive or even negative charge in the region before the hydrophobic stretch could be suggestive of a transmembrane domain rather than a signal peptide, as shown by RmuC and RNAseY sequences described above. Moreover, a clear distinction between transmembrane region and signal peptide might not be critical or not necessarily required to identify factors involved in interaction with the host. Indeed membrane proteins can also bind to host components, as illustrated by Amp, Imp and VmpA (Suzuki et al., 2006; Galetto et al., 2011; Boonrod et al., 2012; Arricau-Bouvery et al., 2018). Thus, it may be wise to include selected candidates for functional studies even if early predictions suggest a N-terminal transmembrane domain (see for instance Strohmayer et al., 2019). And finally, in the case of membrane proteins one could even speculate that post-translational cleavage by proteases other than signal peptidase could release active peptides, similarly to the Tengu and SAP11 effectors which have already been shown to be subjected to such proteolysis (Sugawara et al., 2013; Lu et al., 2014a).

We envision this study as the starting point of a journey in phytoplasma effector biology, of which next stops include, among others, the elucidation of the core- and pan-effectome of phytoplasmas, the identification of effectors evolutive history, and relationships with PMUs. We hope that these results will contribute to a better understanding of phytoplasmas and help pathologists in their quest for phytoplasma virulence factors.

## Data Availability Statement

The datasets presented in this study can be found in online repositories. The names of the repository/repositories and accession number(s) can be found in the article/ Supplementary Material.

## Author Contributions

LB and XF provided the initial idea. CG performed the analyses and drafted the manuscript. All the authors contributed to the article and approved the submitted version.

## Conflict of Interest

The authors declare that the research was conducted in the absence of any commercial or financial relationships that could be construed as a potential conflict of interest.

## Footnotes

1 https://personal.sron.nl/~pault/

2 https://services.healthtech.dtu.dk/service.php?SignalP-4.1

## Supplementary Material

Supplementary Figure 1 Genomic environments of SBP coding sequences from DppA family. SBPs are colored in pink, permease subunits in blue and green, and ATP-binding subunits in orange. Accessions retained in the final DppA dataset are mentioned above schemes and their corresponding coding sequences marked with an asterisk.

Supplementary Figure 2 Alignment of selected sequences from the DppA family showing sequence similarity but different lipoprotein predictions. WP_017192229.1, WP_017193400.1, WP_053521415.1, WP_017192740.1, WP_017191628.1 originate from Milkweed yellows phytoplasma, Vaccinium witches’-broom phytoplasma, *’Candidatus* Phytoplasma pruni’, Italian clover phyllody phytoplasma, Poinsettia branch-inducing phytoplasma respectively. The last four sequences are predicted to contain a type II signal peptide by LipoP (cysteine indicated by an arrow), unlike the first sequence. The five proteins share over 79% of sequence identity between each pair (as calculated by BLASTP) and are expected to act through similar molecular mechanisms, which does not really fit with the hypothesis that the last four could be lipoproteins but not the first one. Residues have been colored according to their biochemical properties up to position 36 of the alignment, and the remaining of the sequences is colored depending on conservation (color codes are the same as Figure 1).

Supplementary Figure 3 Sequence alignment for the AYWB_387 family. The residues have been colored according to biochemical properties up to position 42 of the alignment, and the remaining of the alignment is colored according to conservation between sequences with violet shades.

Supplementary Figure 4 Sequence alignment for the AYWB_376 family. The residues have been colored according to biochemical properties up to position 42 of the alignment, and the remaining of the alignment is colored according to conservation between sequences with violet shades.

Supplementary Figure 5 Sequence alignment for the AYWB_042 family. The residues have been colored according to biochemical properties up to position 65 of the alignment, and the remaining of the alignment is colored according to conservation between sequences with violet shades.

Supplementary Figure 6 Sequence alignment for the RmuC family. The residues have been colored according to biochemical properties up to position 50 of the alignment, and the remaining of the alignment is colored according to conservation between sequences with violet shades.

Supplementary Figure 7 Sequence alignment for the YneF family. The residues have been colored according to biochemical properties up to position 50 of the alignment, and the remaining of the alignment is colored according to conservation between sequences with violet shades.

Supplementary Figure 8 Sequence alignment of the AYWB_444 family. The residues have been colored according to biochemical properties up to position 50 of the alignment, and the remaining of the alignment is colored according to conservation between sequences with violet shades.

Supplementary Figure 9 Sequence alignment for the RNAseY family. The residues have been colored according to biochemical properties up to position 65 of the alignment, and the remaining of the alignment is colored according to conservation between sequences with violet shades. In this alignment, only sequences collected from the nr database of NCBI at the date of the study are shown (accessions listed in Supplementary Material SM15). This includes some of the alternative start codons variants (for example accessions AOF54820.1 and WP_069028241.1 that derive from the same gene of maize bushy stunt phytoplasma strain M3), but not the alternative start codons variants mentioned in Supplementary Material 16.

Supplementary Figure 10 Distribution of signal peptide length for the Tengu, SAP11, SAP54, SBP, AYWB_387, AYWB_376, AYWB_042 and Amp datasets. Redundancy between sequences was addressed by counting only unique signal peptides predicted by each software package.

Supplementary Figure 11 Distribution of prediction scores for all the datasets described in this study. In these plots, each sequence in a given dataset is represented by a dot. Orange dots were assigned to sequences with a positive signal peptide prediction. Sequences that were predicted to have no signal peptide are represented by blue dots.

Supplementary Figure 12 Distribution of cleavage scores for all the datasets described in this study. In these plots, each sequence in a given dataset is represented by a dot. Orange dots were assigned to sequences with a positive signal peptide prediction. Sequences that were predicted to have no signal peptide are represented by blue dots. For SignalP5, sequences that had no predicted signal peptide were assigned a cleavage score of 0.

Supplementary Table 1 Accessions, origin, identity groups, and references for sequences homologous to Tengu.

Supplementary Table 2 Accessions, origin, identity groups, and references for sequences homologous to SAP11.

Supplementary Table 3 Accessions, origin, identity groups, and references for sequences homologous to SAP54.

Supplementary Table 4 SBP accessions used in this study.

Supplementary Table 5 List of sequences associated with NlpA family that are devoid of signal peptides.

Supplementary Table 6 List of phytoplasma SBP gene products predicted to be a lipoprotein according to LipoP.

Supplementary Table 7 Lipoprotein predictions of SBPs from some *Acholeplasma* genomes. This table is adapted from the Supplementary Material 12 of Kube et al. (2014) J. Mol. Microbiol. Biotechnol. 24, 19–36 that recapitulates SBP genes identified in the genomes of *Acholeplasma brassicae*, *A. palmae*, and *A. laidlawii*. Starting from this data, the locus tags were updated using NCBI annotation, and the corresponding protein sequences were submitted to the PredLipo and LipoP servers. The obtained predictions are listed in the columns “PredLipo” and “LipoP.” For better readability, positive lipoproteins predictions have been highlighted in green. The locus tags BN853_RS03515, BN853_RS05710, BN853_RS06865, BN854_RS05460, ACL_RS00205 that were present in the original table but that do not seem to code for SBPs were removed from this table.

Supplementary Table 8 List of accessions used in this study for the AYWB_387, AYWB_376, and AYWB_042 families.

Supplementary Table 9 List of accessions used in this study for RmuC, YneF, AYWB_444, and RNaseY families. One asterisk was added to accessions provided by nr database of NCBI and that derive from genes having alternative start codons. The translation products obtained with the alternative start codons are listed in Supplementary Material SM16. Doubled-asterisk indicate that the accessions derive from the same gene and differ by the start codon used.

Supplementary Table 10 List of sequences from the RNaseY family obtained by using an alternative start codon. These accessions are not part of Genbank.

Supplementary Table 11 List of Amp accessions used in this study.

Supplementary Table 12 Accessions related to VmpA, VmpB, and Vmp1, and the associated signal peptide predictions. Positive signal peptide predictions were indicated with “Y,” and negative ones with “N” and a gray background for enhanced visibility.

Supplementary Table 13 List of Imp accessions used in this study.

Supplementary Material 1 Sequences used in this study, grouped as fasta files.
