# Supplementary material for: Comparison of Current Methods for Signal Peptide Prediction in Phytoplasmas
Source: Front Microbiol. 2021 Mar 25;12:661524. doi: 10.3389/fmicb.2021.661524 (PMC8026896; doi:10.3389/fmicb.2021.661524)
Supplement: Supplementary Figure 1 — Genomic environments of SBP coding sequences from DppA family. SBPs are colored in pink, permease subunits in blue and green, and ATP-binding subunits in orange. Accessions retained in the final DppA dataset are mentioned above schemes and their corresponding coding sequences marked with an asterisk. [file Data_Sheet_1.PDF]

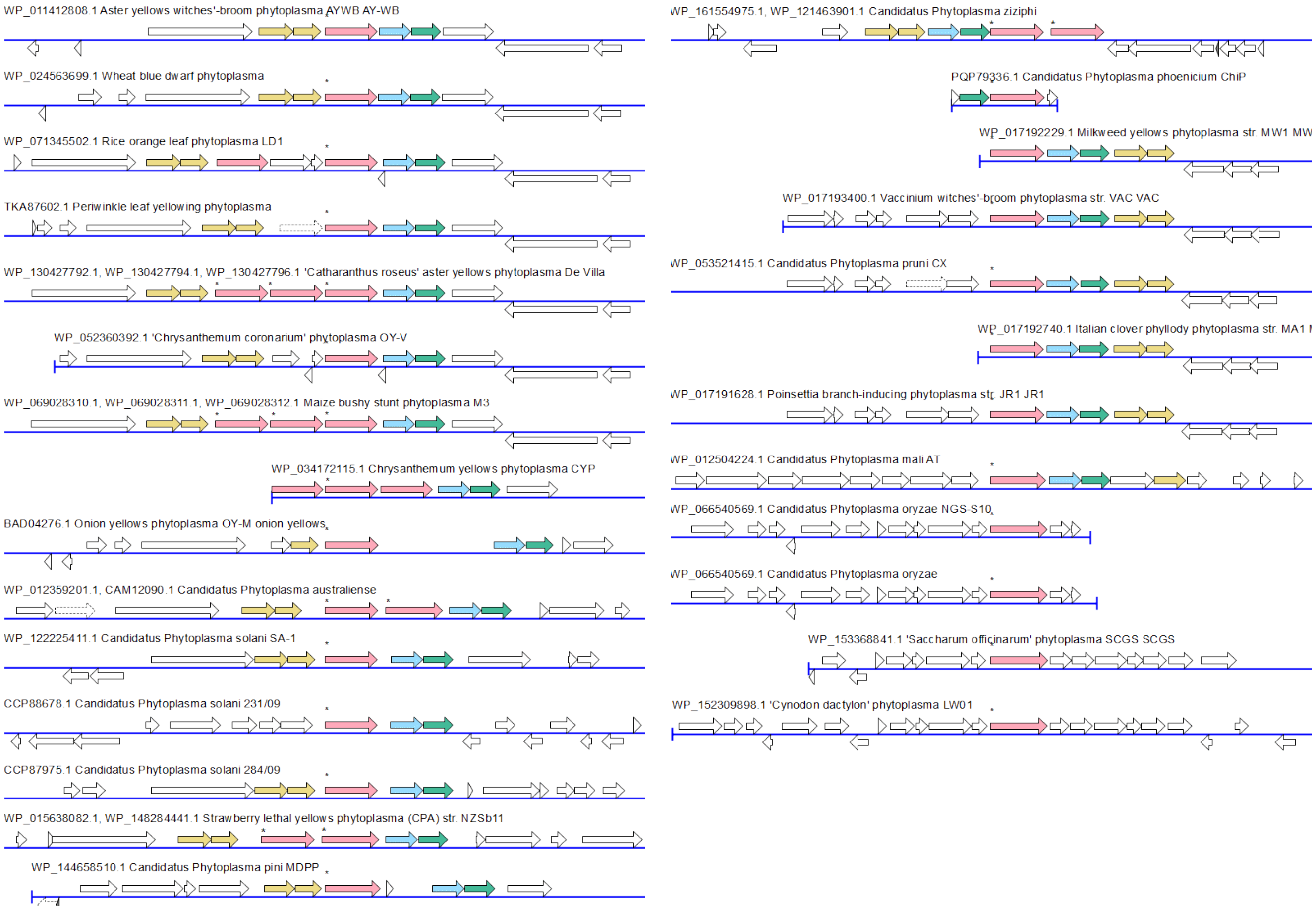

Supplementary Figure S1. Genomic environments of SBP coding sequences from DppA family. SBPs are colored in pink, permease subunits in blue and green, and ATP-binding subunits in orange. Accessions retained in the final DppA dataset are mentioned above schemes and their corresponding coding sequences marked with an asterisk.
