# Supplementary material for: Comparison of Current Methods for Signal Peptide Prediction in Phytoplasmas
Source: Front Microbiol. 2021 Mar 25;12:661524. doi: 10.3389/fmicb.2021.661524 (PMC8026896; doi:10.3389/fmicb.2021.661524)
Supplement: Supplementary Figure 5 — Sequence alignment for the AYWB_042 family. The residues have been colored according to biochemical properties up to position 65 of the alignment, and the remaining of the alignment is colored according to conservation between sequences with violet shades. [file Data_Sheet_5.PDF]

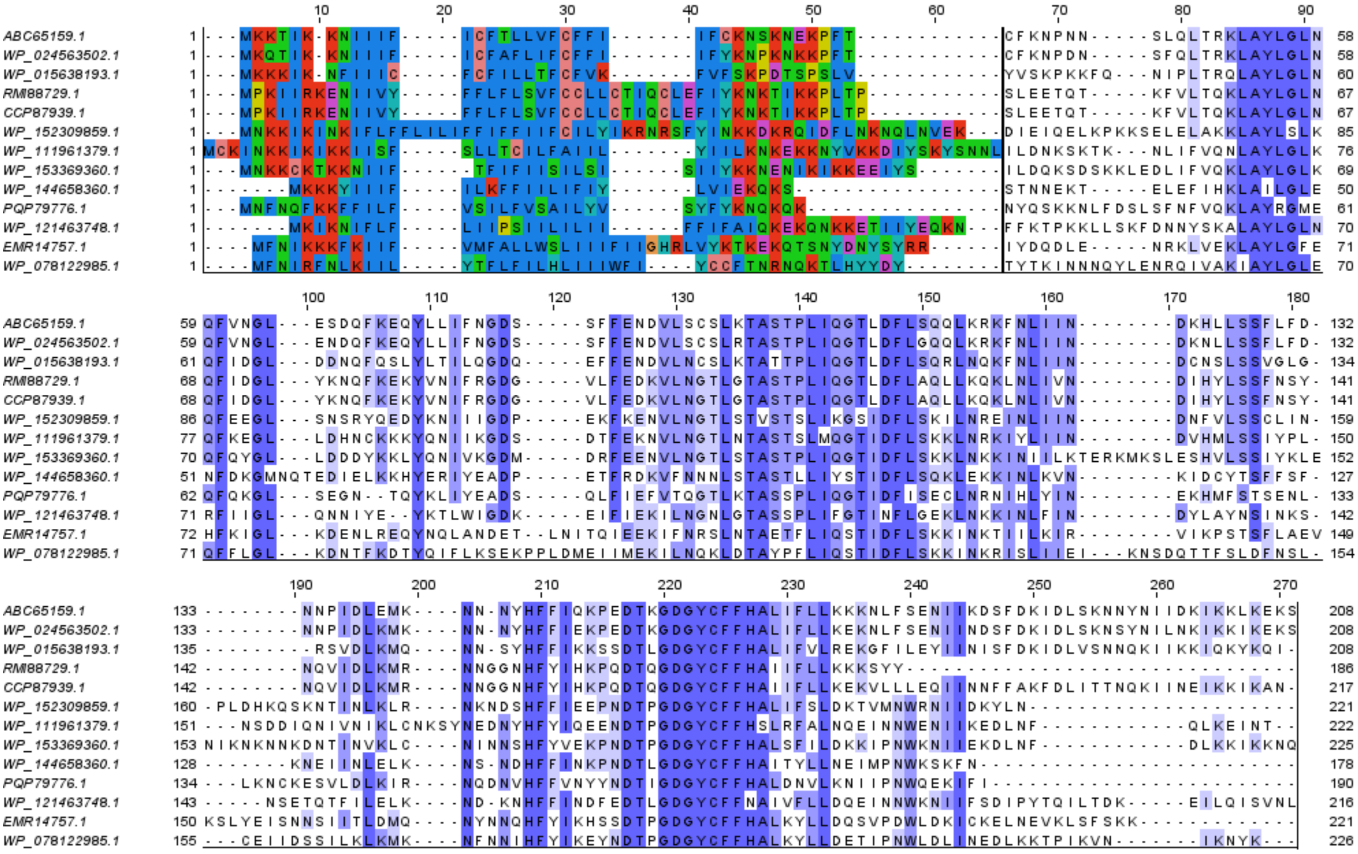

Supplementary Figure S5. Sequence alignment for the AYWB_042 family. The residues have been colored according to biochemical properties up to position 65 of the alignment, and the remaining of the alignment is colored according to conservation between sequences with violet shades.
