# Supplementary material for: Comparison of Current Methods for Signal Peptide Prediction in Phytoplasmas
Source: Front Microbiol. 2021 Mar 25;12:661524. doi: 10.3389/fmicb.2021.661524 (PMC8026896; doi:10.3389/fmicb.2021.661524)
Supplement: Supplementary Figure 7 — Sequence alignment for the YneF family. The residues have been colored according to biochemical properties up to position 50 of the alignment, and the remaining of the alignment is colored according to conservation between sequences with violet shades. [file Data_Sheet_7.PDF]

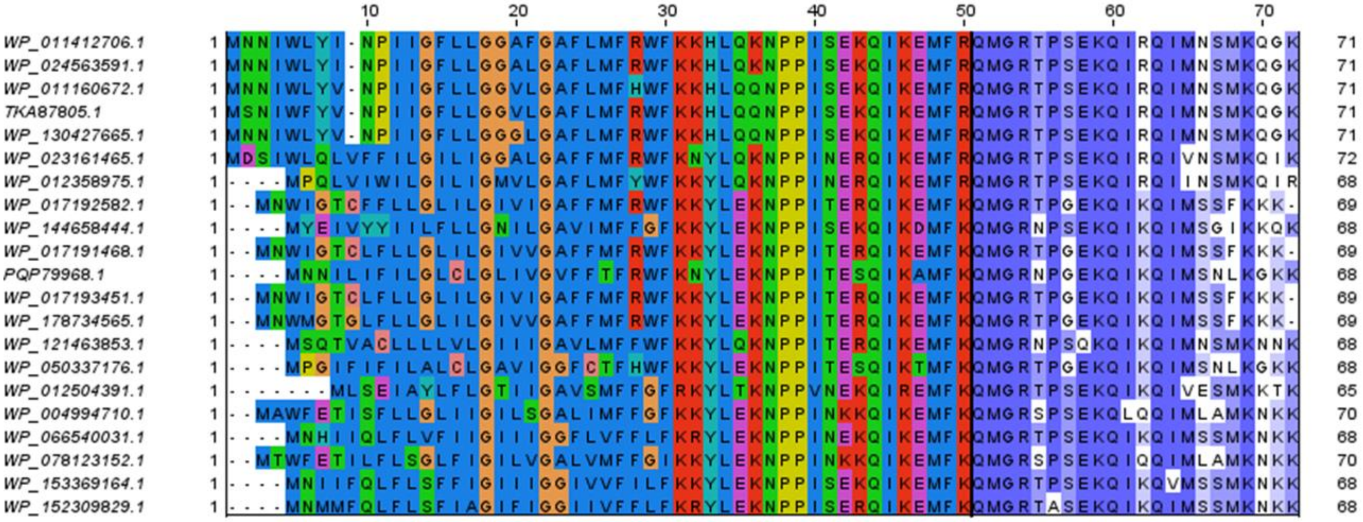

Supplementary Figure S7. Sequence alignment for the YneF family. The residues have been colored according to biochemical properties up to position 50 of the alignment, and the remaining of the alignement is colored according to conservation between sequences with violet shades.
