# Supplementary material for: Comparison of Current Methods for Signal Peptide Prediction in Phytoplasmas
Source: Front Microbiol. 2021 Mar 25;12:661524. doi: 10.3389/fmicb.2021.661524 (PMC8026896; doi:10.3389/fmicb.2021.661524)
Supplement: Supplementary Figure 9 — Sequence alignment for the RNAseY family. The residues have been colored according to biochemical properties up to position 65 of the alignment, and the remaining of the alignment is colored according to conservation between sequences with violet shades. In this alignment, only sequences collected from the nr database of NCBI at the date of the study are shown (accessions listed in Supplementary Material SM15). This includes some of the alternative start codons variants (for example accessions AOF54820.1 and WP_069028241.1 that derive from the same gene of maize bushy stunt phytoplasma strain M3), but not the alternative start codons variants mentioned in Supplementary Material 16. [file Data_Sheet_9.PDF]

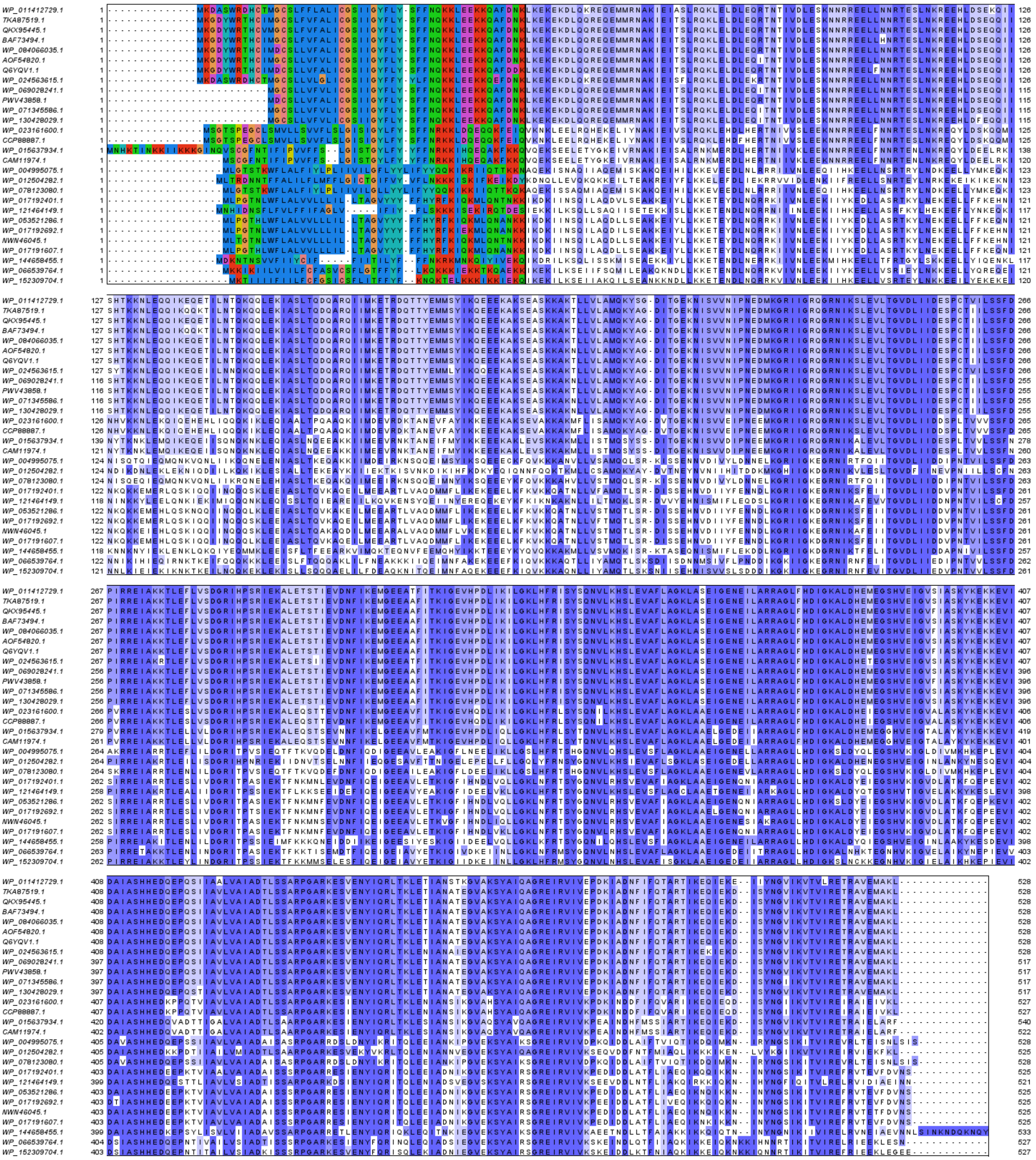

Supplementary Figure S9. Sequence alignment for the RNAseY family. The residues have been colored according to biochemical properties up to position 65 of the alignment, and the remaining of the alignement is colored according to conservation between sequences with violet shades. In this alignment, only sequences collected from the nr database of NCBI at the date of the study are shown (accessions listed in supplemental material SM15). This includes some of the alternative start codons variants (for example accessions AOF54820.1 and WP_069028241.1 that derive from the same gene of maize bushy stunt phytoplasma strain M3), but not the alternative start codons variants mentioned in supplemental material SM16.
