# Supplementary material for: Comparison of Current Methods for Signal Peptide Prediction in Phytoplasmas
Source: Front Microbiol. 2021 Mar 25;12:661524. doi: 10.3389/fmicb.2021.661524 (PMC8026896; doi:10.3389/fmicb.2021.661524)
Supplement: Supplementary Figure 10 — Distribution of signal peptide length for the Tengu, SAP11, SAP54, SBP, AYWB_387, AYWB_376, AYWB_042 and Amp datasets. Redundancy between sequences was addressed by counting only unique signal peptides predicted by each software package. [file Data_Sheet_10.PDF]

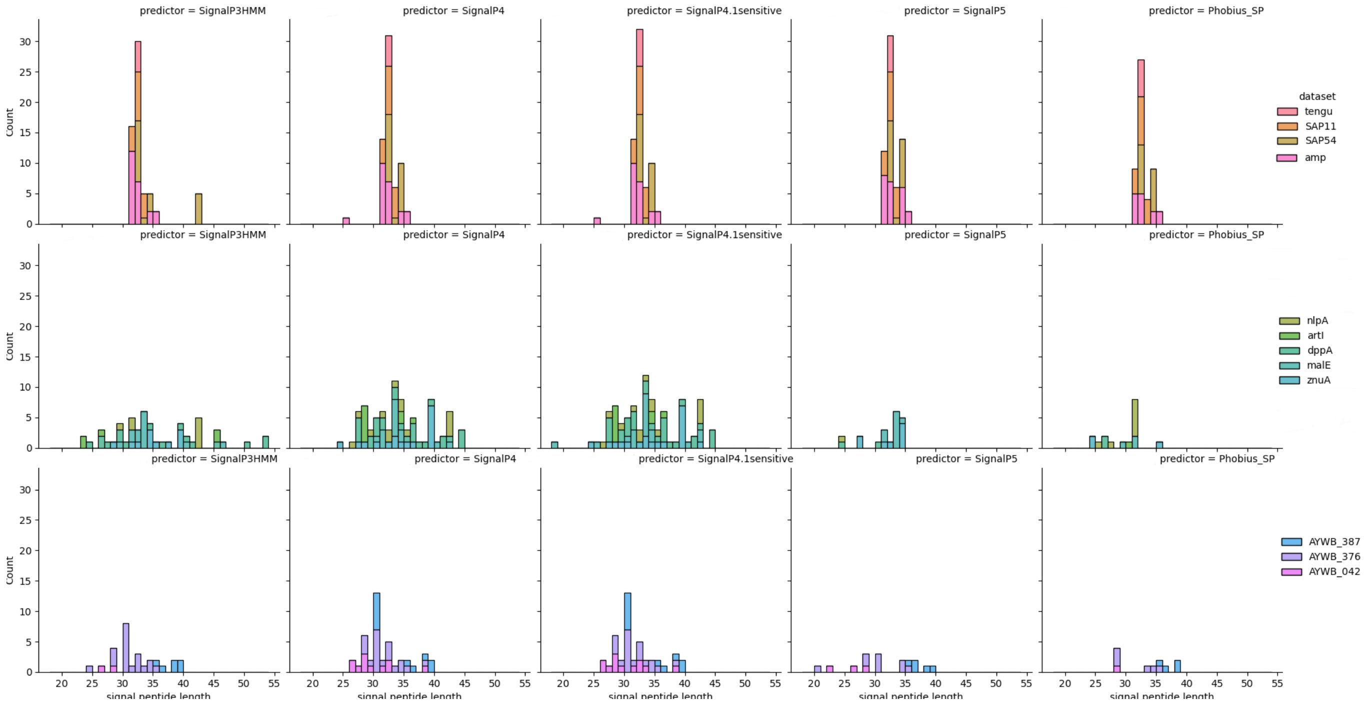

Supplementary Figure S10

Distribution of signal peptide length for the Tengu, SAP11, SAP54, SBP , AYWB_387, AYWB_376, AYWB_042 and Amp datasets. Redundancy beetween sequences was adressed by counting only unique signal peptides predicted by each software package.
