# Supplementary material for: Comparison of Current Methods for Signal Peptide Prediction in Phytoplasmas
Source: Front Microbiol. 2021 Mar 25;12:661524. doi: 10.3389/fmicb.2021.661524 (PMC8026896; doi:10.3389/fmicb.2021.661524)
Supplement: Supplementary Figure 11 — Distribution of prediction scores for all the datasets described in this study. In these plots, each sequence in a given dataset is represented by a dot. Orange dots were assigned to sequences with a positive signal peptide prediction. Sequences that were predicted to have no signal peptide are represented by blue dots. [file Data_Sheet_11.PDF]

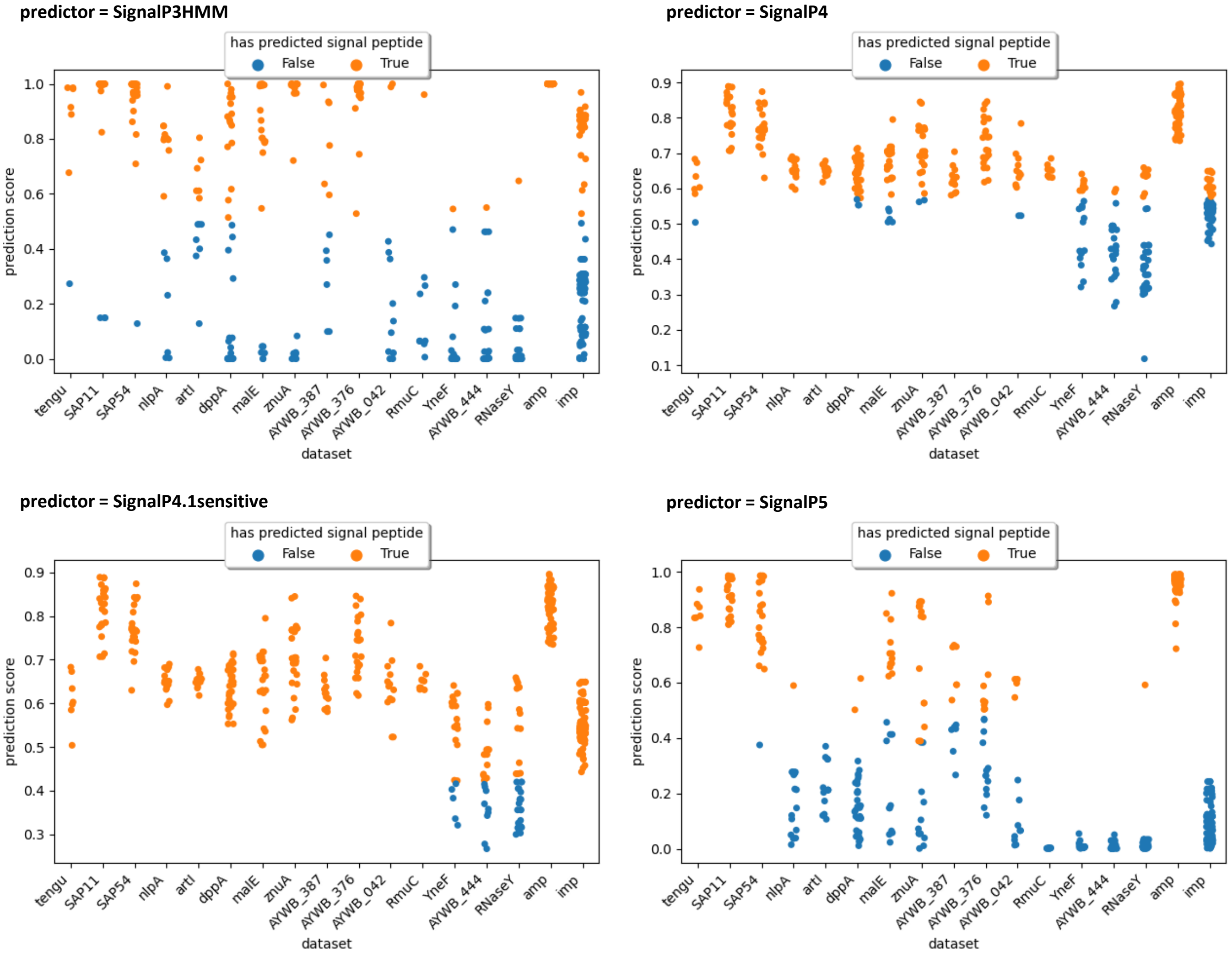

Supplementary Figure S11. Distribution of prediction scores for all the datasets described in this study. In these plots, each sequence in a given dataset is represented by a dot. Orange dots were assigned to sequences with a positive signal peptide prediction. Sequences that were predicted to have no signal peptide are represented by blue dots.
