# Supplementary material for: Comparison of Current Methods for Signal Peptide Prediction in Phytoplasmas
Source: Front Microbiol. 2021 Mar 25;12:661524. doi: 10.3389/fmicb.2021.661524 (PMC8026896; doi:10.3389/fmicb.2021.661524)
Supplement: Supplementary Figure 2 — Alignment of selected sequences from the DppA family showing sequence similarity but different lipoprotein predictions. WP_017192229.1, WP_017193400.1, WP_053521415.1, WP_017192740.1, WP_017191628.1 originate from Milkweed yellows phytoplasma, Vaccinium witches’-broom phytoplasma, ’Candidatus Phytoplasma pruni’, Italian clover phyllody phytoplasma, Poinsettia branch-inducing phytoplasma respectively. The last four sequences are predicted to contain a type II signal peptide by LipoP (cysteine indicated by an arrow), unlike the first sequence. The five proteins share over 79% of sequence identity between each pair (as calculated by BLASTP) and are expected to act through similar molecular mechanisms, which does not really fit with the hypothesis that the last four could be lipoproteins but not the first one. Residues have been colored according to their biochemical properties up to position 36 of the alignment, and the remaining of the sequences is colored depending on conservation (color codes are the same as Figure 1). [file Data_Sheet_2.PDF]

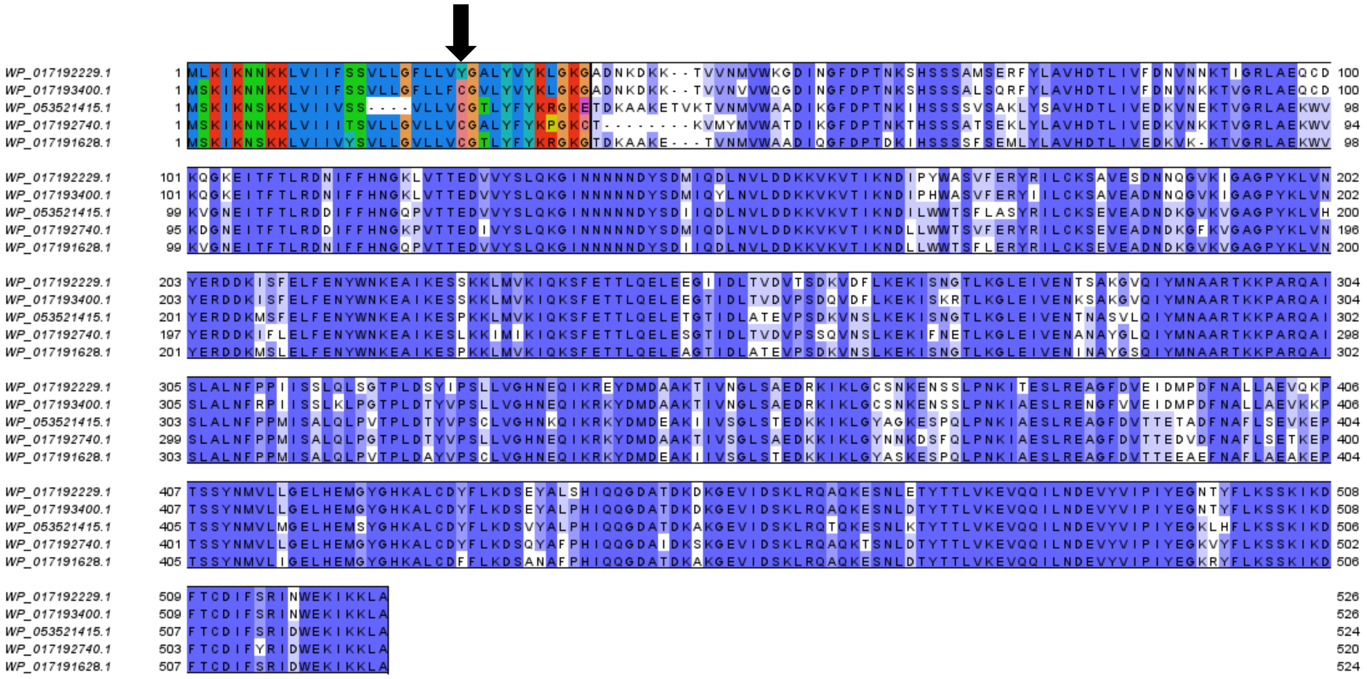

Supplementary Figure S2. Alignement of selected sequences from the DppA family showing sequence similarity but different lipoprotein predictions. WP_017192229.1, WP_017193400.1, WP_053521415.1, WP_017192740.1, WP_017191628.1 originate from Milkweed yellows phytoplasma, Vaccinium witches'-broom phytoplasma, 'Candidatus Phytoplasma pruni', Italian clover phyllody phytoplasma, Poinsettia branch-inducing phytoplasma respectively. The last 4 sequences are predicted to contain a type II signal peptide by LipoP (cysteine indicated by an arrow), unlike the first sequence. The 5 proteins share over 79% of sequence identity between each pair (as calculated by blastp) and are expected to act through similar molecular mechanisms, which does not really fit with the hypothesis that the last 4 could be lipoproteins but not the first one. Residues have been colored according to their biochemical properties up to position 36 of the alignment, and the remaining of the sequences is colored depending on conservation (colour codes are the same as Figure 1).
